# The effects of submission and exposure to real-world and virtual violence during childhood and adolescence

**DOI:** 10.1016/j.jped.2025.101494

**Published:** 2026-01-31

**Authors:** Luci Pfeiffer

**Affiliations:** Universidade Federal do Paraná (UFPR), PhD in Child and Adolescent Health, Curitiba, PR, Brazil

**Keywords:** Children, Adolescents, Exposure to violence, Real world, Virtual world

## Abstract

**Objective:**

This text proposes a discussion and analysis of the effects of exposure to real-world and virtual violence in childhood and adolescence, and its repercussions on development.

**Data sources:**

literature review on the topic of children's and adolescents' exposure to real-world and virtual violence, and observations from case studies of children and adolescents who were victims of serious and very serious violence, assisted by the DEDICA Program – Defense of the Rights of Children and Adolescents in Curitiba in the first semester of 2025.

**Summary of findings:**

It is observed that a significant number of children and adolescents, regardless of their origins, ethnicities, beliefs, cultures, and the sociocultural condition of their guardians, are being exposed to various forms of violence, whether intrafamilial, domestic, institutional, or from their surroundings, through what they observe, witness, and experience in the real or virtual world.

**Conclusions:**

It is concluded that exposure to situations of violence through direct aggression, witnessing, cohabitation, and/or participation can promote desensitization and the normalization of causing harm to others. Without protection or contestation of violent acts, whatever their source, those who are learning their own value and the value of others, and how to fight for life, come to accept them as an inevitable part of life, either suffering them or perpetrating them. The need for pediatric consultations to include guidance for caregivers and patients on preventing exposure to any form of violence, as well as the adoption of protective measures, including legal ones, against the harm to which they may be subjected, is evident.

## Introduction

Children develop physically and psychologically from the care and stimulation they receive, from the transmission of concepts and values, which should include basic limits and prohibitions of protection and respect for oneself and others, from those who will perform the maternal and paternal functions. This learning, progressive and continuous, will occur not only through the word of this other, but also through the attempt to copy the model presented to them by their caregivers, in order to be accepted as an equal.

After birth, the human being goes through a long period of absolute dependence on someone who feeds, cares for, and protects them, who should be stable figures in their life, offering them care, attention, stimulation, and the understanding that they are welcome and sufficient to awaken the affection of this other.^1^

It is the adult caregivers, who are expected to be the parents, the main figures of affection, who will introduce the world to the newborn child and the child to the world, from their first days of life.^2^

Up to the age of seven or eight, depending on the quality and type of affectionate bonds with their caregivers, and what they observe and begin to understand in their surroundings, the child will build the foundations of their personality.^2^^,^^3^ They will retain what is taught directly, but also indirectly, through their impressive capacity for observation and mirroring, to support their primary actions and reactions in dealing with themselves and others, creating the psychological tools with which they will face life and base their choices.

In the absence of a healthy adult role model, sometimes growing up in an environment of mutual aggression between their caregivers and guardians, which is replicated in the most vulnerable, children and adolescents will repeat the place where they managed to survive, whether as victims, hostages of new aggressors, or abusers.

Even in early childhood, the child's gaze turns from their intrafamilial environment to their surroundings. As a recipient of all the influences of the adult world that surrounds them, they expand their sources of knowledge and recognition of their environment, comparing the attention they receive with the different ways in which those who are part of their days, and their actions and reactions, are cared for.

Until adolescence, the child will seek and achieve greater freedom to expand their environments and the view and values ​​of themselves and others, seeking to establish their concepts of right and wrong, of the real, the imaginary and the fantastical, but still within the principles that the adult world around them will be passing on or showing them and what they have access to, whether in the real world or in the virtual world.

The closer, in the physical sense, but especially in terms of bonds of dependence and affection, the earlier, more intense, and longer the child's exposure to situations of violence, the greater the damage to their neuropsychomotor development and to the structuring of their personality, leading to the normalization and naturalization of the suffering imposed by another.

With puberty, the adolescent's gaze and attention will turn, eagerly, in search of new knowledge and values ​​from others, initially from their peers, their families, and those who make up their environment, which will be compared with what they received from their family and are receiving from their nuclear environment. The institutions they attend, such as school and social environments, will play a very important role in presenting and teaching models of right and wrong, and it is expected that they will be healthy in their proposals and examples.

These should be the solid foundations for guaranteeing the rights to the structuring of psychological instruments for respect for oneself and others.

To control and protect from the influence of the environment, the external world, both real and virtual, where violence resides, and social media makes it common news, what has been offered by the family, community, and society in general as a way and model of living and growing for children and adolescents?

### Objectives

The damaging effects of violent environments on the structuring of a child's and adolescent's personality, from birth, are undeniable, as are the formation and deformation of their values, concepts of good or bad living with themselves and with others, leading to greater harm to themselves and society in general, the earlier and longer their exposure to violence lasts.

Under these concepts and principles, this text aims to:

### General objective

To present a study and analysis of the effects of exposure to the various forms of violence that invade everyone's daily life and, consequently, the life and physical and psychological development of children and adolescents, through threats, suffering, witnessing, or involvement with situations of violence, over which there is no command or control in their origins.

This study and analysis raise an alert about the lack of comprehensive epidemiological data to measure the magnitude of the problem in Brazil and the world, and, especially, the need to seek ways and means of protecting children and adolescents.

### *Specific objectives*

• To analyze the main presentations and forms of violence present in Brazilian childhood and adolescence, from intrafamilial to structural, community, and societal violence, in both the real and virtual worlds.
• To raise awareness of the effects of children and adolescents' exposure to direct and indirect forms of violence with which they live.
• To recommend that healthcare professionals who provide care to children and adolescents, especially pediatricians, include in their diagnostic assessments the recognition of signs and symptoms of direct and/or indirect exposure to situations of violence to which they may be subjected, as well as their treatment and the protective measures they need.
• To propose a joint effort with the education, social action, security, and justice sectors to take action to stop this situation, starting by minimizing the exposure of children and adolescents to instances of violence, whether through witnessing, threats, cohabitation, or subjugation, both within the family and originating from the environments in which they live, the communities they frequent, and society in general, disseminated in the real and virtual world.

## Results

Violence has always been part of human history, with children and adolescents being its most vulnerable victims, bearing the brunt of the damage, which multiplies due to the sequential harm to their development.

The WHO emphasizes that violence is the greatest public health and human rights concern in the world. It estimates that 1 billion children – half the world's children – are victims of some type of violence every year. Furthermore, exposure to violence at an early age can impair brain development and cause damage to other parts of the Central Nervous System, as well as the Endocrine, Circulatory, Musculoskeletal, Reproductive, Respiratory and Immune Systems, with lifelong consequences.^4^

In a global context, international organizations such as the UN, WHO and UNICEF have collected alarming data on the prevalence of violence against youth. Regarding violence in schools, UNICEF estimates that half of students aged 13 to 15 —approximately 150 million young people — have been victims of violence by peers inside or outside of school.^5^

Among the age group analyzed, just over one in three students suffers bullying. According to the UN agency, the same proportion is involved in physical fights. In 39 wealthy countries, three out of ten students admit to having bullied their peers. In the short term, this affects their learning and, in the long term, can lead to depression, anxiety, and even suicide. “Violence is an unforgettable lesson that no child needs to learn”.^5^

Regarding violence committed in armed conflicts, between 2005 and 2022, the UN recorded approximately 315,000 serious violations against children in conflict areas, with >120,000 killed or maimed 12. Other violations included the recruitment of 105,000 minors by armed groups, >32,500 kidnappings, and 16,000 cases of sexual violence.^6^

The report from the Brazilian Forum on Public Security (FBSP) points to 44,127 victims of Intentional Violent Deaths (IVD) between 2023 and 2024. IVD is a category that includes intentional homicide, femicide, robbery resulting in death, injury followed by death, and Deaths Resulting from Police Intervention (DRPI).^7^

According to UNICEF reports on the panorama of lethal and sexual violence against children and adolescents in Brazil, 4803 violent deaths of children and adolescents were recorded in 2021, 5354 in 2022, and 4944 in 2023. The majority of victims (91.6 %) were aged between 15 and 19 years old.^8^

### The normalization of violence from the intrafamilial and domestic sphere

Intrafamilial and domestic violence against children and adolescents can be defined as a highly prevalent, chronic, and progressive transgenerational disease that does not depend on ethnicity, creed, or sociocultural situation, with a higher frequency of intrafamilial and domestic origin, which can leave lifelong physical, psychological, and sexual sequelae if not prevented, interrupted, and treated adequately.^9^ It consists of its own signs and symptoms, and in the case of physical and sexual violence, there is also the possibility of diagnostic confirmation through specific examinations.^9^

For those who suffer abuse within their homes, which should be a primary place of care and protection, and a model of life, they grow up in a toxic and disruptive environment. And, learning evil, because, through observation and identification with the adult world that surrounds them, violence can become an effective method for resolving conflicts.

Intrafamily violence committed by those who should have the satisfaction and duty of caring for and protecting their descendants has a triple deleterious effect on childhood and adolescence, both because of direct physical, psychological, and sexual aggression, and because of the loss of developmental potential, with the distortion of the adult model in which they will mirror their way of being and living. As a result, if not prevented and its damage treated early, violence against developing beings, children and adolescents, will determine the inability to fight for a dignified life or the capacity to be cruel.^9^

### The use of violence as consumer material by the media - desensitization, tolerance, saturation and impunity

According to the American Academy of Pediatrics, passive exposure to violence by the media is becoming an uncontrollable component of the lives of children and adolescents.^9^

The media's commercialization of violence, especially in Brazil, illustrates this process well. Research indicates that exposure to violent content, such as news and films, is associated with harmful effects on its viewers, including the effects of post-traumatic stress, such as fear, anxiety, and depression.^10^

In parts of the world, including the studied country, violence, in all its forms, is news that attracts audiences and, due to the increased consumption aimed at profit, has been a topic at any time on television news programs, especially during the so-called "prime time," when families are supposedly gathered together.

Furthermore, it will be available on internet platforms and applications, freely accessible to any age where the use of internet-connected screens is permitted, frequently detailing and showing domestic, urban, or conflict and war scenes that surpass many horror films. They enter the living rooms, bedrooms, and minds of children and adolescents, normalizing violence as something always possible and present, and harm to people of all ages and death as a natural possibility of living, or inevitable and even deserved.

The earlier and more continuous this exposure, the greater the chances of leading to brain alterations and a distorted view of reality.^10^ Depending on the intensity and frequency of this exposure to violence, whether direct or indirect, reactions can occur that may affect the neuropsychomotor development of children and adolescents, leading to alterations in the formation of their psychological instruments for coping with life.

As initial and warning symptoms, it will be possible to identify several signs of psychological suffering, such as non-localized fear, insecurity, anxiety reactions, isolation, phobias, and lack of trust in the world and in life. In the continuation of these excesses, which invade the developing mind, the desensitization and normalization of violent acts may transform them into a natural part of their lives.

### Violence in the virtual world

Unlike the information sought in books, magazines, and newspapers, printed material would be assumed to be authored and the responsibility of someone, and the message would be ready, and the reader could choose whether to be interested in it or reject it before it invaded our vision and minds, and those of children and adolescents.

In the world of screens, what went from a basic and practical means of communication, like the first cell phones offered to people, to an instrument for locating, identifying, analyzing, and trading information about its owners, who no longer determine what is received and shared from this supposedly exclusive property. It has also become a means and instrument for reaching people, and especially a childhood and adolescence unprepared for the good and evil it conveys.

It would not be expected that parents would allow their children free access to screens, and especially to the internet and all its users, access to them. Thus, anyone who has this interest will have access to their stories, routines, preferences, and difficulties, making contact and the offering of content directed to the researched profile an easy and supposedly protected means for all kinds of approaches and harassment. Luckily, the content chosen for this approach may only be for the purpose of stimulating consumption, with financial profit as payment.

According to HAIDT, the transition from basic cell phones to smartphones in the early 2010s intensified and diversified digital activities, exacerbating their four fundamental harms: social deprivation, sleep deprivation, fragmented attention, and addiction.^11^

Safernet Brasil warns about the growing use of Generative Artificial Intelligence in the production of content depicting sexual violence against children, whether through image manipulation or the creation of synthetic content. In 2023, there was a record number of 71,867 reports of some form of violence against children and adolescents.^12^

With the sending and receiving of information, and with the evolution of virtual media and artificial intelligence controlling the interests of those behind the screens, control has been lost, not only regarding privacy, which has ceased to exist, but also due to the invasion of content that encourages access, consumption, and profit from anything that can be negotiated, for anyone, including the amoral and perverse side of humanity, at any price.

Ethics and respect for childhood and adolescence do not seem to exist in parts of the real world and in most virtual environments, and the anonymity that is maintained if there is no legal intervention allows all cybercrimes to evolve in time to cause irreparable damage to this population.

Normalization, therefore, is not just a process of habituation, but video games with a violent basis can lead to cognitive and affective alterations, which increase the predisposition to aggression, the struggle for dominance and power, as well as the distortion of human life values.

Beyond the so-called cybercrimes against childhood and adolescence, which are already quite well-defined and are becoming increasingly present in the lives of millions of children and adolescents, the discussion of which would exceed the scope of this text, there is also the encouragement, teaching, and provision of tools for the practice of devastating violence among peers, such as the so-called "Dangerous Challenges and Games," which, in truth, can become deadly.

The so-called “Dangerous Challenges” can be defined, according to CHAVES GAMA, as the inducement to practice acts and attitudes of extreme risk for those who are led to practice them, disseminated through platforms and applications of the virtual world, especially children and adolescents with some psychological fragility that leads them to believe that they would be games of proof of courage and a way of mistreating another, often presented and disseminated by “influencers” seeking fame at any cost, from the other.^13^

Research from TC Kids Online Brazil, 2024, indicates that, between January and July 2025, SaferNet Brazil National Cybercrime Reporting Channel registered 49,336 anonymous reports of child sexual abuse and exploitation, classified as child pornography, an increase of 18.9 % compared to the same period in 2024. The total number of reports received in 2024 corresponds to 64 % of all notifications received during the period, confirming the centrality of this crime in the Brazilian digital environment.^14^

Data on the frequency and types of use showed that 93 % of the Brazilian population aged 9 to 17 are internet users, with 20,801,145 children and adolescents accessing the internet at least once a day in 12 months. More than eleven million children and adolescents shared text, images, or videos, and 49.1 % of the total watched advertising or publicity that was not appropriate for their age.^14^

The use of smartphones is becoming increasingly early in a child's life, where they learn very quickly how to handle them, but will know nothing about protection and defense against the possibility of being captured by networks of perverse individuals offering them violent content. Through a “fishing” process using artificial intelligence programming, children and adolescents are identified as being connected for long periods without interruption, or late at night or in the early morning, indicating a lack of adult supervision.

As vulnerable targets identified by their internet searches and preferences, content of all kinds, often involving violent practices, including sexual ones, is used to desensitize them and make them easy prey for obtaining material for other aggressive practices, including pornography, and also real scenes of sadomasochism, torture, and terror, among others.

Studies on the effects of children and adolescents' access to violent content, including games, social media, apps, and platforms, lead to conclusions that converge on the same result: the dissemination and propagation of violent material favors, in the short and medium term, the emergence of aggressive feelings at all ages.^15^

Commerce and the consumer industry in the virtual world have created ways to present violence at all levels, for all ages. An example of this induction is "Discord," which, by definition, is a real-time communication platform that functions as a set of virtual communities. It differs from other social networks by being composed of more closed and private servers (groups), where moderation and control can be less rigorous, facilitating the circulation of extreme content.^16^

It hosts violence desensitization and normalization groups, environments for the propagation of hate speech, recruiting and radicalizing young people, leading many to aggressive and antisocial behaviors.^16^

### *Effects of exposure to violence*

According to DESMURGET, the exposure of children and adolescents to all types of proposals and practices of violence presented through news and digital games will determine changes in behavior such as the intensification of aggressive thoughts and behaviors, desensitization, and decreased empathy, and the unfounded exacerbation of the subjective feeling of insecurity.^17^

The violence witnessed is not a passive event, but a traumatic experience that shapes the psyche, behavior, and perception of reality, culminating, in continuity, in a systemic process of normalization. This normalization facilitates violence, as a desensitized public tends to mobilize less against the problem, reinforcing the inaction of people involved, institutions, and powers, perpetuating its cycle.^18^

Intrafamily and domestic violence, the most serious for development, will distort the child's and adolescent's understanding of the aggressions imposed on them, whether physical, psychological, or sexual, leading them to accept them as a natural and deserved form of care and education. They induce the child to accept cruelty against the other, even in a relationship of total dependence and supposed good affection, as "normal" and, therefore, replicable in their other relationships.^2^

Among community violence, urban violence imposes life under threat, and its constant media exposure, as material to attract the audience through horror, makes it commonplace, desensitizing its viewers.

The damage to neuropsychomotor development will be all the more severe the earlier, more intense, and longer the exposure to any type of violence. There is no doubt that the marks of violence will be deeper in intrafamily violence, where the victim is kept as a permanent hostage of their aggressors, from whom they would expect love, care, and protection.^19^

## Conclusions

The suffering, coexistence, witnessing, hearing, and practice, even if imaginative, of any form of violence in childhood and adolescence, phases of greatest neuropsychological development of the human being, can lead to distortions in the vision they are forming of the world and how to live in it.

It would be extremely important that guidance on not exposing children to any type of violence and to screens and the virtual world without strict rules, selection, and supervision of the content to be permanently accessed be included in childcare services, from prenatal care and birth.

The normalization, desensitization, and minimization of the effects resulting from the exposure of children and adolescents to various forms of violence, whether intrafamilial, domestic, community, urban, or global, is a central challenge to be faced in the care and protection of childhood and adolescence.

## Financial support

None declared.

### Data availability statement

The entire dataset supporting the results of this study is available upon request to the corresponding author.

## Conflicts of interest

The author declares no conflicts of interest.
